# The effects of triptolide on the pharmacokinetics of sorafenib in rats and its potential mechanism

**DOI:** 10.1080/13880209.2017.1340963

**Published:** 2017-06-14

**Authors:** Xianming Wang, Xin Zhang, Fei Liu, Minghai Wang, Shiyong Qin

**Affiliations:** Department of General Surgery, Qianfoshan Hospital affiliated to Shandong University, Shandong, China

**Keywords:** CYP3A4, herb–drug interaction, P-gp

## Abstract

**Context:** Combining sorafenib with triptolide could inhibit tumour growth with greater efficacy than single-agent treatment. However, their herb–drug interaction remains unknown.

**Objective:** This study investigates the herb–drug interaction between triptolide and sorafenib.

**Materials and methods:** The effects of triptolide (10 mg/kg) on the pharmacokinetics of different doses of sorafenib (20, 50 and 100 mg/kg) in rats, and blood samples were collected within 48 h and evaluated using LC-MS/MS. The effects of triptolide on the absorption and metabolism of sorafenib were also investigated using Caco-2 cell monolayer model and rat liver microsome incubation systems.

**Results:** The results showed that the *C*_max_ (low dose: 72.38 ± 8.76 versus 49.15 ± 5.46 ng/mL; medium dose: 178.65 ± 21.05 versus 109.31 ± 14.17 ng/mL; high dose: 332.81 ± 29.38 versus 230.86 ± 9.68 ng/mL) of sorafenib at different doses increased significantly with the pretreatment of triptolide, and while the oral clearance rate of sorafenib decreased. The *t*_1/2_ of sorafenib increased significant (*p* < 0.05) from 9.02 ± 1.16 to 12.17 ± 2.95 h at low dose with the pretreatment of triptolide. Triptolide has little effect on the absorption of sorafenib in Caco-2 cell transwell model. However, triptolide could significantly decrease the intrinsic clearance rate of sorafenib from 51.7 ± 6.37 to 32.4 ± 4.43 μL/min/mg protein in rat liver microsomes.

**Discussion and conclusions:** These results indicated that triptolide could change the pharmacokinetic profiles of sorafenib in rats; these effects might be exerted via decreasing the intrinsic clearance rate of sorafenib in rat liver.

## Introduction

Sorafenib is an orally active tyrosine kinase inhibitor, which has been approved for the treatment of renal and hepatocellular carcinoma (Abdel-Rahman 2013; Abdulghani et al. 2013; Ahmadi et al. 2014). Research indicates that sorafenib could inhibit tumour cell proliferation by targeting Raf/mitogen-activated protein kinase or extracellular signal-regulated kinase, and exert antiangiogenic effects by targeting vascular endothelial growth factor receptor-1/-2/-3 and platelet-derived growth factor receptor-b tyrosine kinases (Bhatt and Ganti 2014; Booth et al. 2014; Brown et al. 2014; Chen et al. 2014).

In humans, sorafenib is administrated in a tablet form, and the majority (77%) of the sorafenib dose is either not absorbed or is eliminated through the hepatobiliary route (50% unchanged), whereas 19% of the dose (mostly glucuronides) is excreted in urine (Lathia et al. 2006; Kim et al. 2015; Schmithals et al. 2015; Shimada et al. 2015). Some studies have investigated its intestine absorption and hepatobiliary disposition to enhance its absorption and clinical efficacy (Wang et al. 2011; Hornecker et al. 2012; Tlemsani et al. 2015; Wang et al. 2015). Recently, some research articles have indicated that the combination of sorafenib and triptolide is superior to single-drug treatment in increasing cell death and apoptosis, and combining sorafenib with triptolide could also inhibit tumour growth with greater efficacy than single-agent treatments (Alsaied et al. 2014). As we know, the absorption of sorafenib in small-intestinal mucosa was mainly mediated by P-glycoprotein (P-gp), and sorafenib was mainly metabolized by cytochrome P450 3A4 (CYP3A4) and uridine diphosphate glucuronosyl transferase 1A9 (UGT1A9) (Doi et al. 2011; Zimmerman et al. 2012; Gillani et al. 2014). Therefore, the drug–drug interaction of sorafenib mediated by CYP3A4 and P-gp might happen. To the best of our knowledge, there is little data available for the effects of triptolide on the pharmacokinetics of sorafenib.

In this study, the herb–drug interaction potential of between sorafenib and triptolide was investigated. *In vivo*, the pharmacokinetics of sorafenib with and without pretreatment of triptolide was determined using a sensitive and reliable LC-MS/MS method. The *in vitro* effects of triptolide on the absorption of sorafenib were investigated in the Caco-2 cell transwell model, and the effects of triptolide on the metabolic stability of sorafenib were also determined using rat liver microsomes.

## Materials and methods

### Chemicals and reagents

Sorafenib (purity >98%), β-nicotinamide adenine dinucleotide phosphate (NADP) and Lucifer yellow was purchased from Sigma (St. Louis, MO). Berberine (purity >98%) and triptolide (purity >98%) were purchased from the National Institute for the Control of Pharmaceutical and Biological Products (Beijing, China). Rat liver microsomes were purchased from BD GentestTM (Becton Dickinson, Franklin Lakes, NJ). The Alkaline Phosphatase Assay Kit was provided by Nanjing Jiancheng Bioengineering Institute (Nanjing, China). Acetonitrile and methanol were purchased from Fisher Scientific (Fair Lawn, NJ). Formic acid was purchased from Anaqua Chemicals Supply Inc. Limited (Houston, TX). Ultrapure water was prepared with a Milli-Q water purification system (Millipore, Billerica, MA). All other chemicals were of analytical grade or better.

Dulbecco’s modified Eagle’s medium (DMEM) and non-essential amino acid (NEAA) solution were purchased from Thermo Scientific Corp. (Logan, UT). Foetal bovine serum (FBS) was obtained from GIBCO BRL (Grand Island, NY). Penicillin G (10,000 U/mL) and Streptomycin (10 mg/mL) were purchased from Amresco (Solon, OH). Hanks' balanced salt solution (HBSS) was purchased from GIBCO (Grand Island, NY).

### Animal experiments

Sprague–Dawley (SD) rats (including equal numbers of males or females) weighing 230–250 g were provided by the Experimental Animal Center of the Shandong University (Jinan, China). Rats were bred in a breeding room at 25 °C, 60 ± 5% humidity, and a 12 h dark–light cycle. Tap water and normal chow were given *ad libitum*. All the experimental animals were housed under the above conditions for a 3-d acclimation and were fasted overnight before the experiments. The animal facilities and protocols were approved by the Institutional Animal Care and Use Committee. All procedures were in accordance with the National Institute of Health guidelines regarding the principles of animal care.

### *In vivo* pharmacokinetic study

To evaluate the effects of triptolide on the pharmacokinetics of sorafenib, the rats were divided into six groups of six animals each. Sorafenib was dissolved in 0.9% saline (the final concentration of sorafenib was 2, 5 and 10 mg/mL, respectively) and orally administered to rats at different dose (20, 50 and 100 mg/kg, respectively). The pretreated groups received oral triptolide (10 mg/kg) 30 min prior to sorafenib. Triptolide was dissolved in normal saline containing PEG400 (1:20) at a concentration of 20 mg/mL. Blood samples (0.25 mL) were collected into a heparinized tube via the *oculi chorioideae* vein at 0.25, 0.5, 1, 2, 4, 6, 8, 12, 24, 36 h and 48 h. The blood samples were centrifuged at 3500 rpm for 10 min, and the plasma samples obtained were stored at −40 °C until analysis.

### LC-MS/MS method for the determination of sorafenib in rat plasma

The LC-MS/MS method was performed according to our previously reported (Wang et al. 2015). The analysis was performed on an Agilent 1290 series liquid chromatography system (Agilent Technologies, Palo Alto, CA), including a binary pump, an on-line vacuum degasser, a surveyor autosampling system, a column temperature controller, and an Agilent 6460 triple-quadruple mass spectrometer (Agilent Technologies, Palo Alto, CA) with Turbo Ion spray, which is connected to the liquid chromatography system. Agilent MassHunter B 4.0 software (Agilent Technologies, Palo Alto, CA) was used for the control of equipment and data acquisition. The chromatographic analysis of sorafenib was performed on Waters X-Bridge C18 column (3.0 × 100 mm, i.d.; 3.5 μm, Waters Co., Milford, MA) at room temperature. The mobile phase was water (containing 0.1% formic acid) and acetonitrile (62:38, v:v) at a flow rate of 0.4 mL/min.

The mass scan mode was positive MRM mode. The precursor ion and the product ion are *m*/*z* 465.1 → 252.2 for sorafenib and *m*/*z* 336.2 → 320.2 for IS, respectively. The collision energy for sorafenib and IS was 30 eV and 25 eV, respectively. The MS/MS conditions were optimized as follows: fragmentor, 130 V; capillary voltage, 4 kV; nozzle voltage, 500 V; nebulizer gas pressure (N_2_), 40 psig; drying gas flow (N_2_), 10 L/min; gas temperature, 350 °C; sheath gas temperature, 400 °C; sheath gas flow, 12 L/min.

### Data analysis

The pharmacokinetic parameters, including the area under the plasma concentration–time curve (*AUC*), the maximal plasma concentration (*C*_max_), the time for maximal plasma concentration (*T*_max_), and the mean residence time (*MRT*) were calculated using the DAS 3.0 pharmacokinetic software (Chinese Pharmacological Association, Anhui, China).

The differences between the mean values were analyzed for significance using one-way analysis of variance (ANOVA). *p* Values less than 0.05 were considered as statistically significant.

### Cell culture

The Caco-2 cell line was obtained from the American Type Culture Collection (Manassas, VA). The Caco-2 cells were cultured in DMEM high-glucose medium containing 15% FBS, 1% NEAA and 100 U/mL penicillin and streptomycin. The cells were cultured at 37 °C with 5% CO_2_. For transport studies, the cells at passage 40 were seeded on transwell polycarbonate insert filters (1.12 cm^2^ surface, 0.4 μm pore size, 12 mm diameter; Corning Costar Corporation, Cambridge, MA) in 12-well plates at a density of 1 × 10^5^ cells/cm^2^. Cells were allowed to grow for 21 d. For the first 7 d, the medium was replaced every 2 d, and then daily. The transepithelial electrical resistance (TEER) of the monolayer cells was measured using Millicell ERS-2 (Millipore Corporation, Billerica, MA), and TEER exceeding 400 Ω cm^2^ was used for the flux experiment. The integrity of the Caco-2 monolayers was confirmed by the paracellular flux of Lucifer yellow, which was less than 1% per hour. The passive diffusion was validated using a transcellular transport marker propranolol (10 μM), and the efflux activity of P-gp was validated using a typical *P-gp* substrate digoxin (25 μM). The qualified monolayers were used for transport studies.

### Effects of triptolide on the transport of sorafenib in the Caco-2 cell transwell model

Before the transport experiments, the cell monolayers were rinsed twice using warm (37 °C) Hanks’ balanced salt solution (HBSS), and then the cells were incubated at 37 °C for 20 min. After preincubation, the cell monolayers were incubated with sorafenib in fresh incubation medium from either the apical or basolateral side for the indicated times at 37 °C. The volume of incubation medium on the apical and basolateral sides was 0.5 mL and 1.5 mL, respectively, and a 100 μL aliquot of the incubation solution was withdrawn at the indicated time points from the receiver compartment and replaced with the same volume of fresh pre-warmed HBSS buffer. The effects of triptolide on the transport of sorafenib were investigated by adding 20 μM triptolide to both sides of the cell monolayers and preincubating the sample for 30 min at 37 °C. The permeability of sorafenib (2 μM) in all of the above conditions for both directions, i.e., from the apical (AP) side to the basolateral (BL) side or from the BL side to the AP side, was measured after incubation for 2 h at 37 °C.

The apparent permeability coefficient (*P*_app_) was calculated by using the equation of Artursson and Karlsson:

$$P_{app}=\left(\Delta \text{Q}/\Delta \text{t}\right)\times \left[\text{1}/\left(\text{A}\times {\text{C}}_{0}\right)\right]$$

where *P*_app_ is the apparent permeability coefficient (cm/s), Δ*Q*/Δ*t* (μmol/s) is the rate at which the compound appears in the receiver chamber, *C*_0_ (μmol/L) is the initial concentration of the compound in the donor chamber and *A* (cm^2^) represents the surface area of the cell monolayer. Data were collected from three separate experiments, and each was performed in triplicate.

### Effects of triptolide on the metabolic stability of sorafenib in rat liver microsomes

Rat liver microsomes were used to determine the phase I metabolic stability of sorafenib. The assay conditions and reaction mixtures were similar as reported previously with minor modification (Qi et al. 2013; Li et al. 2016). In brief, except for NADPH-generating system (10 mM G-6-P, 1 mM NADP^+^, 4 mM magnesium chloride, 1 unit/mL of G-6-PDH), 10 μL rat liver microsome (20 mg/mL), 4 μL sorafenib solution (100 μM) and 366 μL PBS buffer (0.1 M, pH 7.4) were added to the centrifuge tubes on ice. The reaction mixture was incubated at 37 °C for 5 min and then NADPH-generating system (15 μL) was added. The effects of triptolide on the metabolic stability of sorafenib was investigated by adding 100 μM triptolide to rat liver microsomes and preincubating for 30 min at 37 °C, and then NADPH-generating system (15 μL) was added. Aliquots of 30 μL were collected from reaction volumes at 0, 1, 3, 5, 15, 30 and 60 min and 60 μL ice-cold acetonitrile containing IS was added to terminate the reaction, and then the sample preparation method was the same as the plasma sample preparation method and determined by LC-MS/MS.

The *in vitro* half-life (*t*_1/2_) was obtained using the equation: *t*_1/2_ = 0.693/k; *V* (μL/mg) = volume of incubation (μL)/protein in the incubation (mg); intrinsic clearance (Clint) (μL/min/mg protein) = *V*× 0.693/*t*_1/2_.

## Results and discussion

### Effects of triptolide on the pharmacokinetics of sorafenib

**Figure 1.**
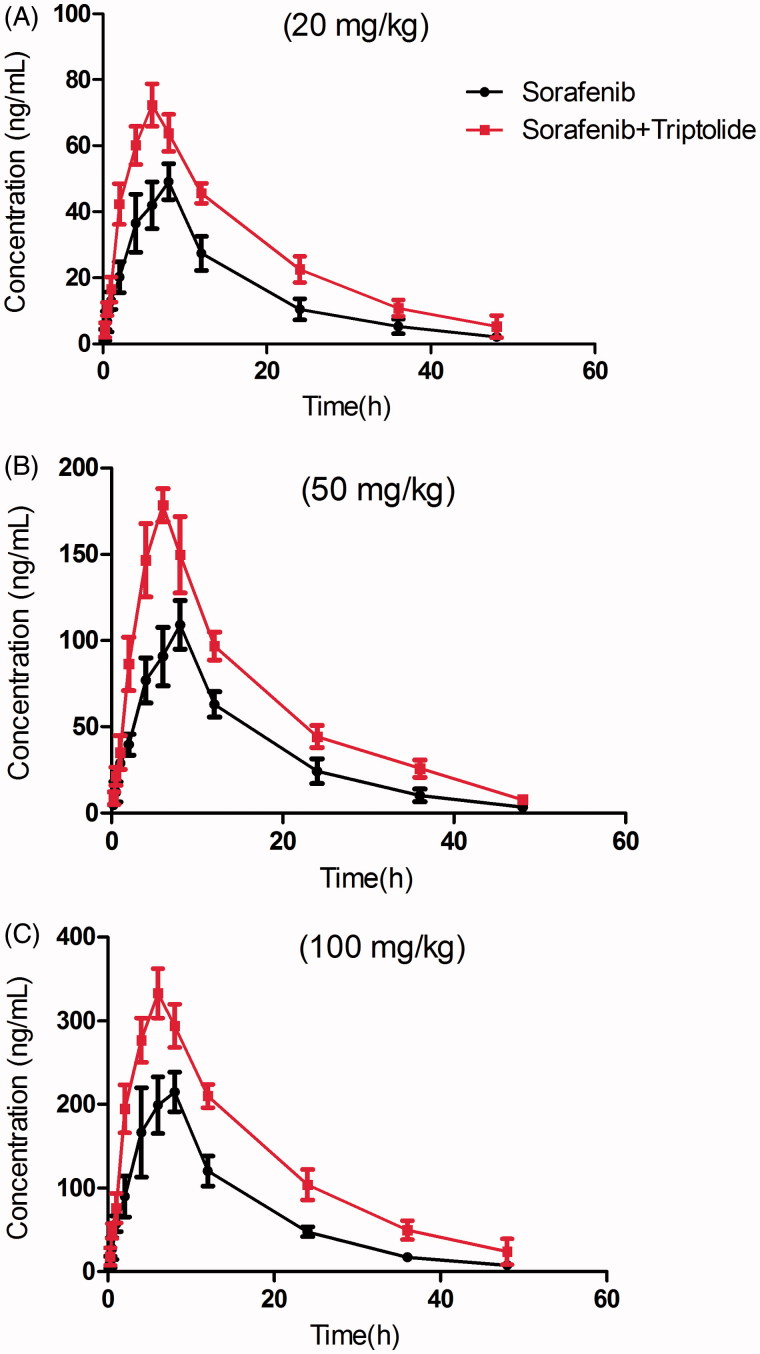
The pharmacokinetic profiles of sorafenib in rats after oral administration of different dose of sorafenib (20, 50 and 100 mg/kg) with or without treatment of triptolide (10 mg/kg). (A) Dose of sorafenib at 20 mg/kg; (B) dose of sorafenib at 50 mg/kg; (C) dose of sorafenib at 100 mg/kg.

The mean plasma concentration–time curves of sorafenib after oral administration of sorafenib or both sorafenib and triptolide are presented in Figure 1.

The pharmacokinetic parameters of sorafenib were calculated using the non-compartmental method with DAS 3.0 pharmacokinetic software (Chinese Pharmacological Association, Anhui, China). The pharmacokinetic parameters are shown in Table 1.

**Table 1.** The pharmacokinetic parameters of sorafenib in rats after oral administration of different dose of sorafenib (20, 50 and 100 mg/kg; *n* = 6, mean ± S.D.) with or without treatment of triptolide.

	Low dose (20 mg/kg)		Medium dose (50 mg/kg)		High dose (100 mg/kg)	
Parameter	Control	Test	Control	Test	Control	Test
*T*_max_ (h)	8.05 ± 1.18	6.16 ± 0.95	8.10 ± 0.95	6.08 ± 0.79	8.14 ± 1.05	6.10 ± 0.88
*C*_max_ (μg/L)	49.15 ± 5.46	72.38 ± 8.76*	109.31 ± 14.17	178.65 ± 21.05*	230.86 ± 9.68	332.81 ± 29.38*
*t*_1/2_ (h)	9.02 ± 1.16	12.17 ± 2.95*	9.40 ± 0.97	11.35 ± 3.97	8.90 ± 1.31	11.02 ± 2.32
*AUC*_(0_*_*–t*_*_)_ (μg h/L)	768.91 ± 157.37	1335.12 ± 174.94*	1689.73 ± 291.89	2931.03 ± 294.30*	3361.68 ± 348.59	6141.31 ± 804.75*
*AUMC*_(0_*_*–t*_*_)_ (μg h/L)	10591.89 ± 2263.23	20094.27 ± 3720.15*	22922.62 ± 5115.52	42697.04 ± 5324.61*	44357.38 ± 4409.07	92433.32 ± 11226.34*
Oral CL (L/h/kg)	25.87 ± 5.36	14.09 ± 2.78*	29.21 ± 5.28	16.22 ± 2.45*	29.08 ± 3.17	15.32 ± 2.53*
*MRT* (h)	13.65 ± 2.27	15.07 ± 2.55	13.48 ± 1.75	14.55 ± 0.47	13.21 ± 0.77	14.98 ± 0.98

* *p* < 0.05 indicate significant differences from the control.

The results showed that the *C*_max_ and *AUC*_(0_*_–t_*_)_ of sorafenib at different doses increased significantly, and while the oral clearance rate of sorafenib decreased with the pretreatment of triptolide. The *t*_1/2_ of sorafenib increased from 9.02 ± 1.16 to 12.17 ± 2.95 h at low dose with the pretreatment of triptolide, and the difference was significant (*p* < 0.05). The *t*_1/2_ of sorafenib also increased at medium and high dose, but the difference was not significant.

These results indicated that when the rats were pretreated with triptolide, the plasma concentration of sorafenib was significantly increased. We think that after pretreatment with triptolide, *P-gp* might be inhibited by triptolide and that the absorption of sorafenib was thus increased, as sorafenib was a substrate of *P-gp*. Additionally, sorafenib has been reported to be metabolized by CYP3A4 in liver, and therefore, we propose that triptolide might also cause the higher *C*_max_ and lower oral clearance rate of sorafenib by inhibiting CYP3A-mediated metabolism. Therefore, to validate this proposition, Caco-2 cell transwell experiment and rat liver microsome incubation experiment were conducted.

### Effects of triptolide on the bidirectional transport of sorafenib across Caco-2 cells

To investigate the effects of triptolide on the transport of sorafenib, the Caco-2 cell *in vitro* model was utilized. First, the transport of 2 μM of sorafenib across Caco-2 cell monolayers was investigated. The *P*_appAB_ and *P*_appBA_ were 6.97 ± 1.08 × 10^−6^ and 1.82 ± 0.52 × 10^−5 ^cm/s, respectively. The *P*_appBA_ was much higher than *P*_appAB_, and the efflux ratio was 2.61, which indicated that efflux transporters might be involved in the transport of sorafenib. Then the transport studies were performed in the presence of 20 μM triptolide to determine the effects of triptolide on the transport of sorafenib. In the presence of 20 μM of triptolide, the *P*_app_ values from the AP side to the BL side increased from 6.97 ± 1.08 × 10^−6^ to 7.13 ± 1.22 × 10^−6 ^cm/s, whereas those from the BL side to the AP side decreased from 1.82 ± 0.52 × 10^−5^ to 1.71 ± 0.34 × 10^−6 ^cm/s. The efflux ratio decreased from 2.61 to 2.39, and the difference was not significant. These results indicated that triptolide had little influence on the transport of sorafenib.

### Effects of triptolide on the metabolic stability of sorafenib in rat liver microsomes

The effects of triptolide on the metabolic stability of sorafenib were investigated using rat liver microsomes. The metabolic half-life of sorafenib was 26.8 ± 3.65 min, and the intrinsic clearance rate was 51.7 ± 6.37 μL/min/mg protein. While the metabolic half-life was prolonged (42.8 ± 5.82 min) in the presence of triptolide, and the intrinsic clearance rate (32.4 ± 4.43 μL/min/mg protein) was increased. These results indicated that triptolide could slow down the metabolism of sorafenib in rat liver microsomes, which might work through inhibiting the activity of CYP3A4 to affect the metabolism of sorafenib.

## Conclusions

In this study, the herb–drug interaction between sorafenib and triptolide were investigated using LC-MS/MS, and the results indicated that triptolide could change the pharmacokinetics profiles of sorafenib. These effects might be exerted via decreasing the intrinsic clearance rate of sorafenib in rat liver via inhibiting the activity of CYP3A4. Consequently, the herb–drug interaction between triptolide and sorafenib might occur when triptolide and sorafenib were co-administered.

## References

[CIT0001] Abdel-Rahman O. 2013 Systemic therapy for hepatocellular carcinoma (HCC): from bench to bedside. J Egypt Natl Canc Inst. 25:165–171. 24207088 10.1016/j.jnci.2013.08.002

[CIT0002] Abdulghani J, Allen JE, Dicker DT, Liu YY, Goldenberg D, Smith CD, Humphreys R, El-Deiry WS. 2013 Sorafenib sensitizes solid tumors to Apo2L/TRAIL and Apo2L/TRAIL receptor agonist antibodies by the Jak2-Stat3-Mcl1 axis. PLoS One. 8:e75414. 24086526 10.1371/journal.pone.0075414 PMC3784419

[CIT0003] Ahmadi M, Ahmadihosseini Z, Allison SJ, Begum S, Rockley K, Sadiq M, Chintamaneni S, Lokwani R, Hughes N, Phillips RM. 2014 Hypoxia modulates the activity of a series of clinically approved tyrosine kinase inhibitors. Br J Pharmacol. 171:224–236. 24117380 10.1111/bph.12438 PMC3874709

[CIT0004] Alsaied OA, Sangwan V, Banerjee S, Krosch TC, Chugh R, Saluja A, Vickers SM, Jensen EH. 2014 Sorafenib and triptolide as combination therapy for hepatocellular carcinoma. Surgery. 156:270–279. 24953273 10.1016/j.surg.2014.04.055

[CIT0005] Bhatt VR, Ganti AK. 2014 Sorafenib in squamous cell carcinoma of the head and neck: molecular basis and potential role. Future Oncol. 10:17–20. 10.2217/fon.13.212 24328406

[CIT0006] Booth LA, Tavallai S, Hamed HA, Cruickshanks N, Dent P. 2014 The role of cell signalling in the crosstalk between autophagy and apoptosis. Cell Signal. 26:549–555. 24308968 10.1016/j.cellsig.2013.11.028 PMC4054685

[CIT0007] Brown WS, Khalili JS, Rodriguez-Cruz TG, Lizee G, McIntyre BW. 2014 B-Raf regulation of integrin alpha-4-beta-1-mediated resistance to shear stress through changes in cell spreading and cytoskeletal association in T cells. J Biol Chem. 289:23141–23153. 24936068 10.1074/jbc.M114.562918 PMC4132812

[CIT0008] Chen Y, Huang Y, Reiberger T, Duyverman AM, Huang P, Samuel R, Hiddingh L, Roberge S, Koppel C, Lauwers GY, et al 2014 Differential effects of sorafenib on liver versus tumor fibrosis mediated by stromal-derived factor 1 alpha/C-X-C receptor type 4 axis and myeloid differentiation antigen-positive myeloid cell infiltration in mice. Hepatology. 59:1435–1447. 24242874 10.1002/hep.26790 PMC3966948

[CIT0009] Doi N, Tomita M, Hayashi M. 2011 Absorption enhancement effect of acylcarnitines through changes in tight junction protein in Caco-2 cell monolayers. Drug Metab Pharmacokinet. 26:162–170. 21206134 10.2133/dmpk.dmpk-10-rg-071

[CIT0010] Gillani TB, Rawling T, Murray M. 2014 Cytochrome P450-mediated biotransformation of sorafenib and its n-oxide metabolite: implications for cell viability and human toxicity. Chem Res Toxicol. 28:92–102. 25489883 10.1021/tx500373g

[CIT0011] Hornecker M, Blanchet B, Billemont B, Sassi H, Ropert S, Taieb F, Mir O, Abbas H, Harcouet L, Coriat R, et al 2012 Saturable absorption of sorafenib in patients with solid tumors: a population model. Invest New Drugs. 30:1991–2000. 22006162 10.1007/s10637-011-9760-z

[CIT0012] Kim A, Widemann BC, Krailo M, Jayaprakash N, Fox E, Weigel B, Blaney SM. 2015 Phase 2 trial of sorafenib in children and young adults with refractory solid tumors: a report from the Children's Oncology Group. Pediatr Blood Cancer. 62:1562–1566. 26207356 10.1002/pbc.25548 PMC4515771

[CIT0013] Lathia C, Lettieri J, Cihon F, Gallentine M, Radtke M, Sundaresan P. 2006 Lack of effect of ketoconazole-mediated CYP3A inhibition on sorafenib clinical pharmacokinetics. Cancer Chemother Pharmacol. 57:685–692. 16133532 10.1007/s00280-005-0068-6

[CIT0014] Li H, Liu L, Xie L, Gan D, Jiang X. 2016 Effects of berberine on the pharmacokinetics of losartan and its metabolite EXP3174 in rats and its mechanism. Pharm Biol. 54:2886–2894. 27327872 10.1080/13880209.2016.1190762

[CIT0015] Qi XY, Liang SC, Ge GB, Liu Y, Dong PP, Zhang JW, Wang AX, Hou J, Zhu LL, Yang L, et al 2013 Inhibitory effects of sanguinarine on human liver cytochrome P450 enzymes. Food Chem Toxicol. 56:392–397. 23500771 10.1016/j.fct.2013.02.054

[CIT0016] Schmithals C, Koberle V, Korkusuz H, Pleli T, Kakoschky B, Augusto EA, Ibrahim AA, Arencibia JM, Vafaizadeh V, Groner B, et al 2015 Improving drug penetrability with iRGD leverages the therapeutic response to sorafenib and doxorubicin in hepatocellular carcinoma. Cancer Res. 75:3147–3154. 26239478 10.1158/0008-5472.CAN-15-0395

[CIT0017] Shimada M, Okawa H, Kondo Y, Maejima T, Kataoka Y, Hisamichi K, Maekawa M, Matsuura M, Jin Y, Mori M, et al 2015 Monitoring serum levels of sorafenib and its n-oxide is essential for long-term sorafenib treatment of patients with hepatocellular carcinoma. Tohoku J Exp Med. 237:173–182. 26477611 10.1620/tjem.237.173

[CIT0018] Tlemsani C, Huillard O, Arrondeau J, Boudou-Rouquette P, Cessot A, Blanchet B, Thomas-Schoemann A, Coriat R, Durand JP, Giroux J, et al 2015 Effect of glucuronidation on transport and tissue accumulation of tyrosine kinase inhibitors: consequences for the clinical management of sorafenib and regorafenib. Expert Opin Drug Metab Toxicol. 11:785–794. 25809423 10.1517/17425255.2015.1030392

[CIT0019] Wang X, Zhang X, Huang X, Li Y, Wu M, Liu J. 2015 The drug–drug interaction of sorafenib mediated by P-glycoprotein and CYP3A4. Xenobiotica. 18: 1–8. 10.3109/00498254.2015.1109160 26582036

[CIT0020] Wang XQ, Fan JM, Liu YO, Zhao B, Jia ZR, Zhang Q. 2011 Bioavailability and pharmacokinetics of sorafenib suspension, nanoparticles and nanomatrix for oral administration to rat. Int J Pharm. 419:339–346. 21843612 10.1016/j.ijpharm.2011.08.003

[CIT0021] Zimmerman EI, Roberts JL, Li L, Finkelstein D, Gibson A, Chaudhry AS, Schuetz EG, Rubnitz JE, Inaba H, Baker SD. 2012 Ontogeny and sorafenib metabolism. Clin Cancer Res. 18:5788–5795. 22927483 10.1158/1078-0432.CCR-12-1967 PMC3490489

